# Publisher Correction: Environmental eustress modulates β-ARs/CCL2 axis to induce anti-tumor immunity and sensitize immunotherapy against liver cancer in mice

**DOI:** 10.1038/s41467-021-26376-8

**Published:** 2021-10-14

**Authors:** Chaobao Liu, Yang Yang, Cheng Chen, Ling Li, Jingquan Li, Xiaonan Wang, Qiao Chu, Lin Qiu, Qian Ba, Xiaoguang Li, Hui Wang

**Affiliations:** 1 State Key Laboratory of Oncogenes and Related Genes, Center for Single-Cell Omics, School of Public Health, Shanghai Jiao Tong University School of Medicine, Shanghai, 200025 China; 2 CAS Key Laboratory of Nutrition, Metabolism and Food safety, Shanghai Institute of Nutrition and Health, University of Chinese Academy of Sciences, Chinese Academy of Sciences, Shanghai, China; 3 School of Basic Medical Sciences, Fudan University, Shanghai, 200032 China

**Keywords:** Cancer microenvironment, Liver cancer, Liver

Correction to: *Nature Communications* 10.1038/s41467-021-25967-9, published online 30 September 2021.

In this article Jingquan Li was incorrectly denoted as being one of the equally contributing authors. The original article has been corrected. Following the publication of the original article, it was noted that, due to a typesetting error, the figure labelling for Figure 6A-G was incorrect in the Results section “EE overcomes PD-L1 based checkpoint blockade resistance”. The PDF and HTML versions of the Article have been corrected.

