# Supplementary material for: Environmental eustress modulates β-ARs/CCL2 axis to induce anti-tumor immunity and sensitize immunotherapy against liver cancer in mice
Source: Nat Commun. 2021 Sep 30;12:5725. doi: 10.1038/s41467-021-25967-9 (PMC8484272; doi:10.1038/s41467-021-25967-9)
Supplement: Supplementary file 1 — Supplementary information [file 41467_2021_25967_MOESM1_ESM.pdf]

Supplementary information

Environmental eustress modulates β-ARs/CCL2 axis to induce antitumor immunity and sensitize immunotherapy against liver cancer in mice

List of contents

Supplementary Fig.1: Environmental eustress reduces chemical or HFD induced HCC tumorigenesis

Supplementary Fig.2: Effect of environmental eustress on tumor growth and infiltration of immune cells in the tumor microenvironment

Supplementary Fig.3: Environmental eustress abolishes the immunosuppressive effect of G-MDSC and M2 macrophages on $CD8^+$ T cells

Supplementary Fig.4: CCL2 is required for the EE induced antitumor immunity

Supplementary Fig.5: EE enhances SNS/β-ARs signaling to inhibit HCC tumorigenesis and progression

Supplementary Fig.6: Blockade of SNS/β-ARs signaling abolishes EE-induced CCL2 reduction in tumor cells and immune cells

Supplementary Fig.7: Environmental eustress enhances tumor β-AR signaling to augment the therapeutic of PD-L1/PD-1 blockade

Supplementary Fig.8: ADRB1, ADRB2 and ADRB3 mRNA expression in human liver cancer

Supplementary Fig.9: ADRB1, ADRB2 and ADRB3 mRNA expression in multiple types of human tumor and adjacent normal tissues

Supplementary Fig.10: Higher expression of ADRB1, ADRB2 and ADRB3 expression related to better prognosis in HCC patients

Supplementary Fig. 11: Example of gating schematics to characterize the infiltrated immune cell in tumor tissue

Supplementary Table 1: Reagents used in this study.

Supplementary Table 2: Antibodies used in this study.

Supplementary Table 3: Primers used in this study.

Supplementary Figures

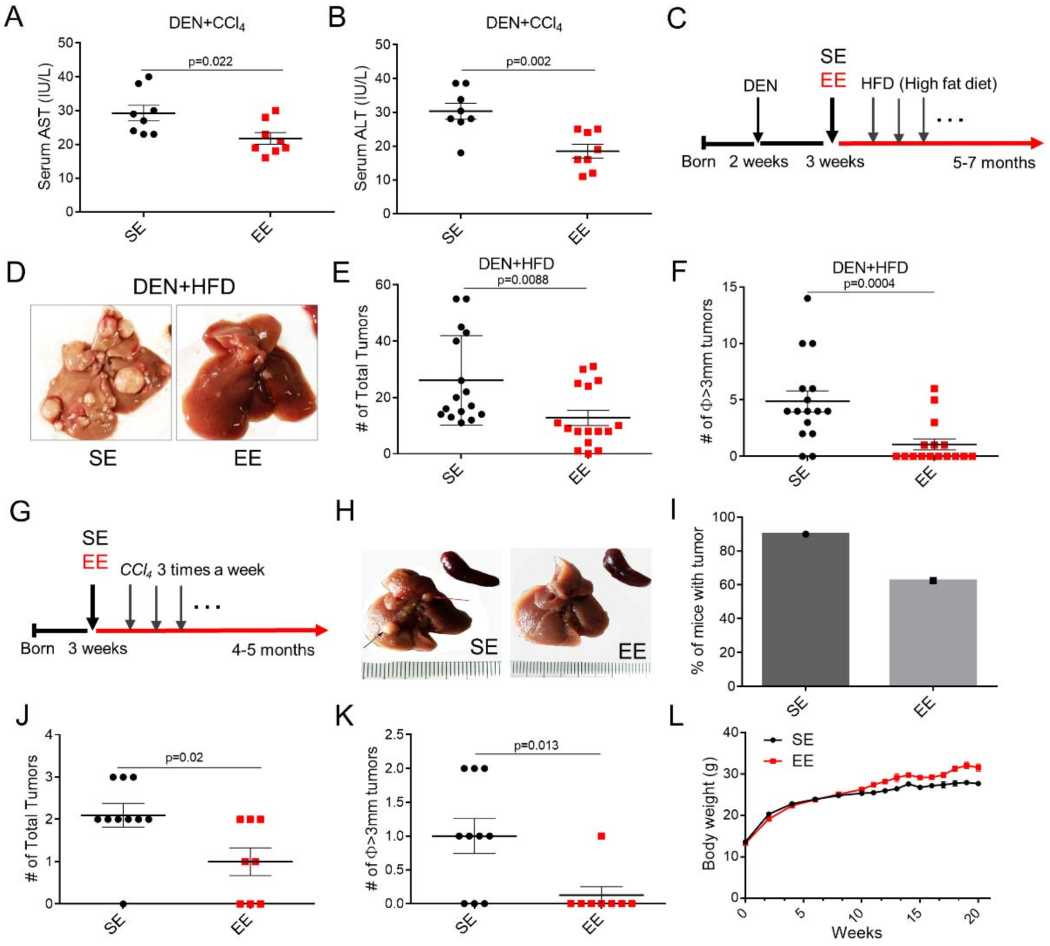

Supplementary Fig.1, related to Fig.1. Environmental eustress reduces chemical or HFD induced HCC tumorigenesis

(A, B) ELISA analysis of AST (A) and ALT (B) in serum from tumor-bearing mice injected with DEN+CCl_4 under SE or EE feeding conditions (n=8). All data are presented as the mean ± SEM, and analyzed by two-tailed unpaired Student's *t* test.(C) Schematic representation of the experimental design for DEN/HFD-induced tumor model, which includes one injection of DEN (25 mg/kg, i.p.) at 2 weeks old, HFD feeding at 3 weeks old under SE or EE feeding conditions, and analysis of tumor numbers and size at 5-7 months old. HFD, high-fat diet.

(D) Representative images of liver for DEN+HFD-induced HCC tumor model with SE or EE feeding conditions.

(E, F) Total tumor number (E) and diameter ø≥3mm tumor number (F) in mice treated with DEN+HFD under SE or EE feeding conditions (n=16). All data are presented as the mean ±

SEM, and analyzed by two-tailed unpaired Student's *t* test. (G) Schematic representation of the experimental design for CCl_4-induced HCC tumor model, which including continuous injections of CCl_4 (0.5 ml/kg, three times per week) at 3 weeks old with SE or EE feeding conditions, and tumor analysis at 4-5months old.

(H) The representative image of liver and spleen from mice in CCl_4-indcued tumor model with SE or EE feeding conditions. Arrowheads indicated the tumor nodules.

(I-K) The proportion of mice with tumor burden (I), total tumor numbers (J) and numbers of tumors with diameter ø⩾3mm (K) in CCl_4 induced HCC mouse models (SE: n=8, EE: n=10). All data are presented as the mean ± SEM, and analyzed by two-tailed unpaired Student's *t* test.(L) Body weight changes of mice injected in Fig. S1G model.

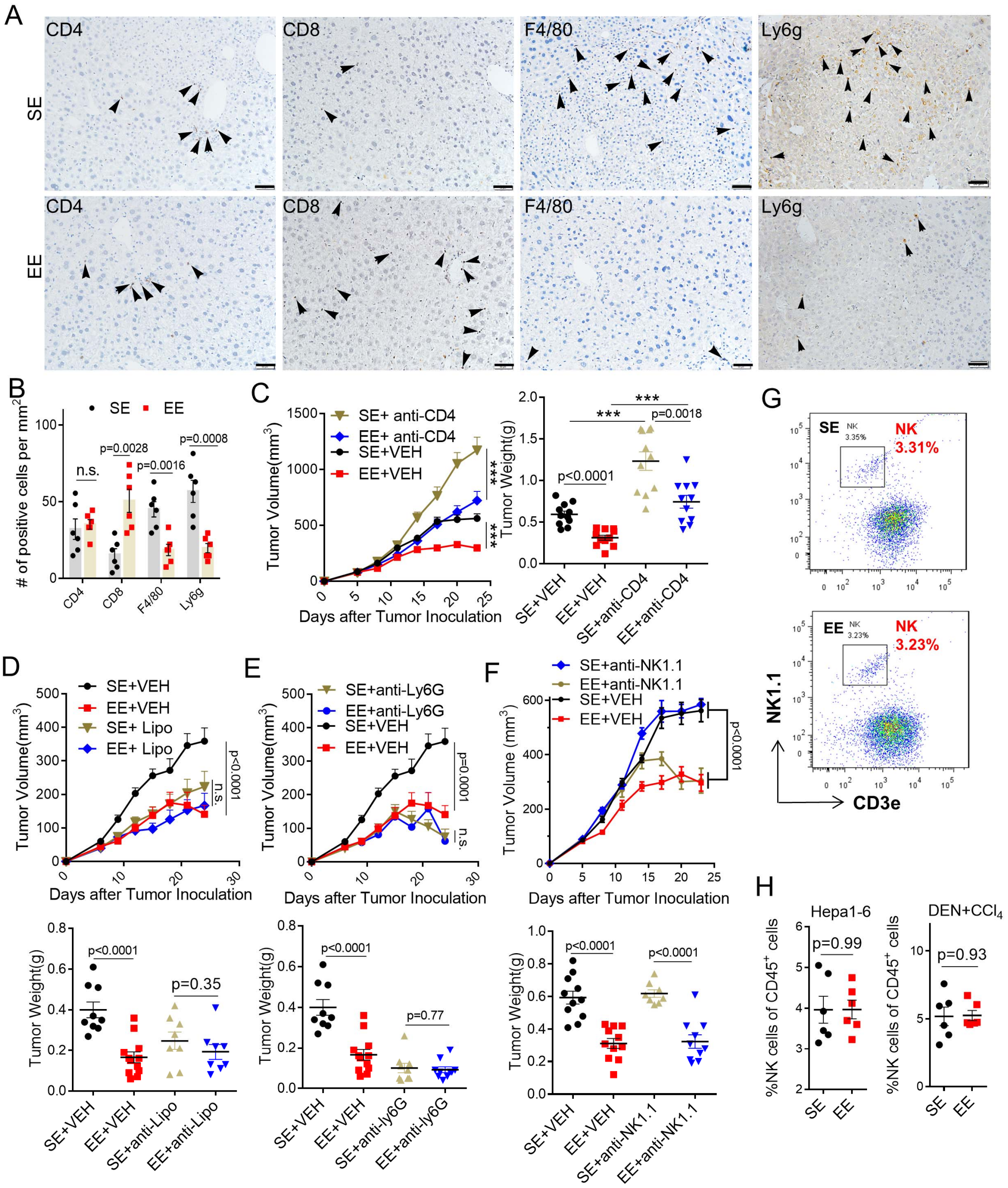

Supplementary Fig.2, related to Fig.2. Effect of environmental eustress on tumor growth and infiltration of immune cells in the tumor microenvironment.

(A) Immunostaining of CD4, CD8, F4/80, and Ly6G on liver tumor tissues from DEN+CCl_4–induced HCC model under SE or EE feeding conditions. Original magnification 20 x 10, Scale bar, 50 μm.

(B) Positive areas of CD4, CD8, F4/80, and Ly6G on liver tumors tissues area were quantified by ImageJ analysis in DEN+CCl_4-induced HCC mouse model (n=6). All data are presented as the mean ± SEM, and analyzed by two-tailed unpaired Student's *t* test with $*p<0.05$, $**p<0.01$, $***p<0.001$.(C) Tumor volume and tumor weight of subcutaneous Hepa1-6 tumors in

C57BL/6 mice treated with anti-CD4 neutralization antibody or vehicle under SE or EE feeding condition (n=11). All data are presented as the mean ± SEM, and analyzed by two-tailed unpaired Student's *t* test with $*p<0.05$, $**p<0.01$, $***p<0.001$.(D) Tumor volume and tumor weight of subcutaneous Hepa1-6 tumors in C57BL/6 mice with macrophages depletion (treated with clodronate liposomes, SE+VEH: n=9, EE+VEH n=12, SE+Lipo: n=8, EE+Lipo: n=8). All data are presented as the mean ± SEM, and analyzed by two-tailed unpaired Student's *t* test.(E) Tumor volume and tumor weight of subcutaneous Hepa1-6 tumors in C57BL/6 mice with G-MDSC depletion (treated with anti-Ly6G neutralization antibody, SE+VEH: n=9, EE+VEH: n=12, SE+anti-Ly6G: n=9, EE+anti-Ly6G: n=10). All data are presented as the mean ± SEM, and analyzed by two-tailed unpaired Student's *t* test.

(F) Tumor volume and tumor weight of subcutaneous Hepa1-6 tumors in C57BL/6 mice with NK cells depletion (treated with anti-NK1.1 neutralization antibody, SE+VEH: n=11, EE+VEH: n=11, SE+anti-NK1.1: n=8, EE+anti-NK1.1: n=10). All data are presented as the mean ± SEM, and analyzed by two-tailed unpaired Student's *t* test.

(G) Representative flow cytometry gating images show the percentage of NK cells in the tumor microenvironment of DEN+CCl_4-induced HCC mice under SE or EE housing condition.

(H) The proportion of NK cells in the mice tumor microenvironment under SE or EE housing condition from DEN+CCl_4 induced tumor model and Hepa1-6 tumor model (n=6). All data are presented as the mean ± SEM, and analyzed by two-tailed unpaired Student's *t* test.

Mean ± SEM, Hepa1-6 n.s. p=0.99, DEN+CCl_4 n.s. p=0.93 by two-tailed unpaired Student's *t* test.

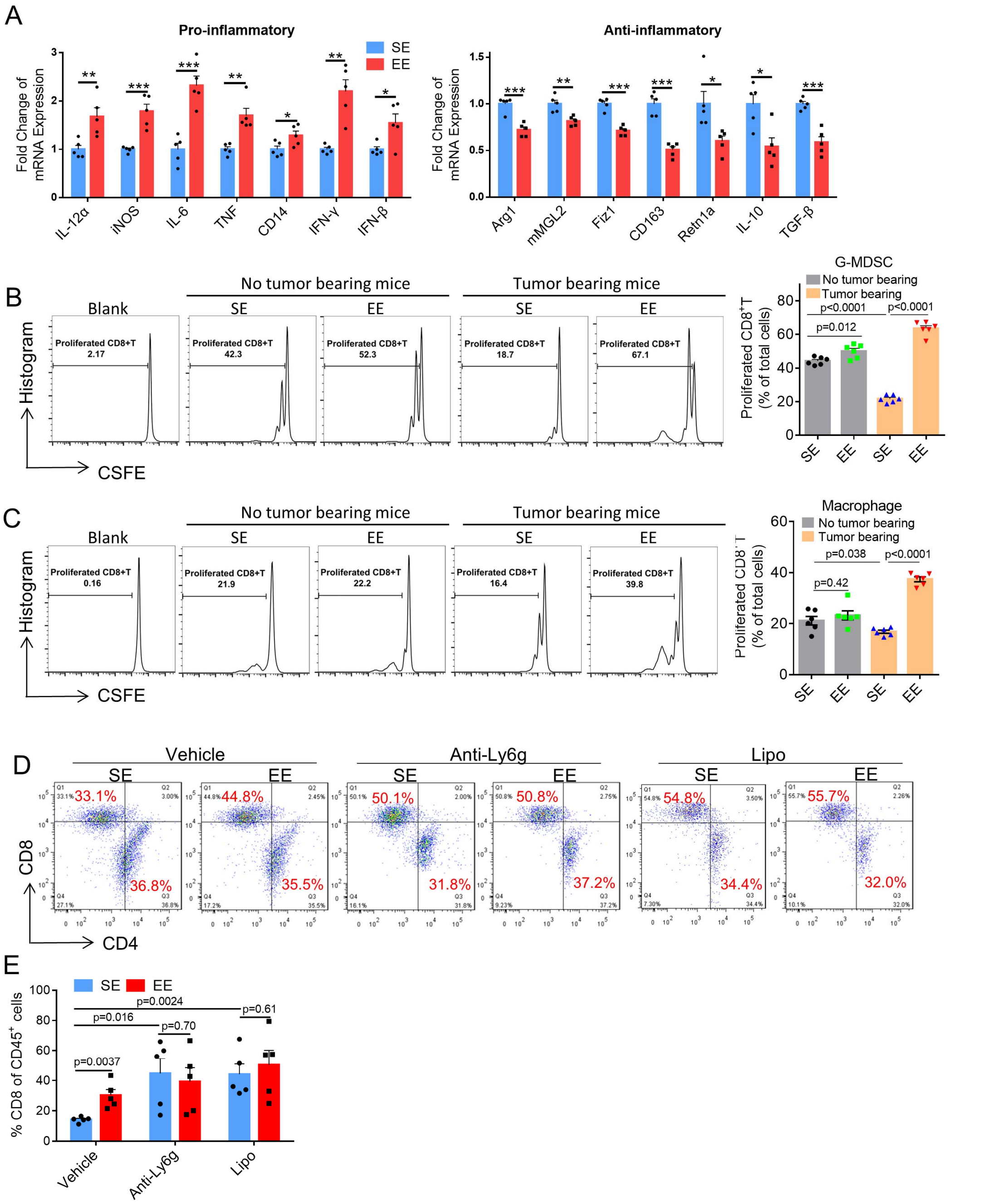

Supplementary Fig.3, related to Fig.2. Environmental eustress abolishes the immunosuppressive effect of G-MDSC and M2 macrophages on CD8$^+$ T cells.

(A) mRNA expressions of typical pro-inflammatory and anti-inflammatory markers were quantified by qRT-PCR assay in subcutaneous Hepa1-6 tumors under SE or EE feeding condition (n=5). All data are presented as the mean ± SEM, and analyzed by two-tailed unpaired Student's *t* test with $*p<0.05$, $**p<0.01$, $***p<0.001$.(B, C) Bone marrow-derived cells were isolated from the femurs of normal mice or subcutaneous Hepa1-6 tumor-burden mice under SE or EE feeding condition. Bone marrow-derived cells were further polarized into G-MDSC by GM-CSF (B) or M2 macrophages by M-CSF and IL-4/IL-13 treatment (C). CD8$^+$ T cells isolated from normal mice spleen were labeled with CFSE and cocultured with

G-MDSC or M2 macrophages for 48h with the stimulation of IL-2, anti-CD3, and anti-CD28 antibody. The proliferation rates of $CD8^+$ T cells were detected by flow cytometry (n=6). All data are presented as the mean ± SEM, and analyzed by two-tailed unpaired Student's *t* test.(D, E) The proportion of $CD8^+$ T cells were analyzed by flow cytometry in subcutaneous Hepa1-6 tumor microenvironment with macrophages or G-MDSC depletion from Fig.S2D and E models (n=5). All data are presented as the mean ± SEM, and analyzed by two-tailed unpaired Student's *t* test.

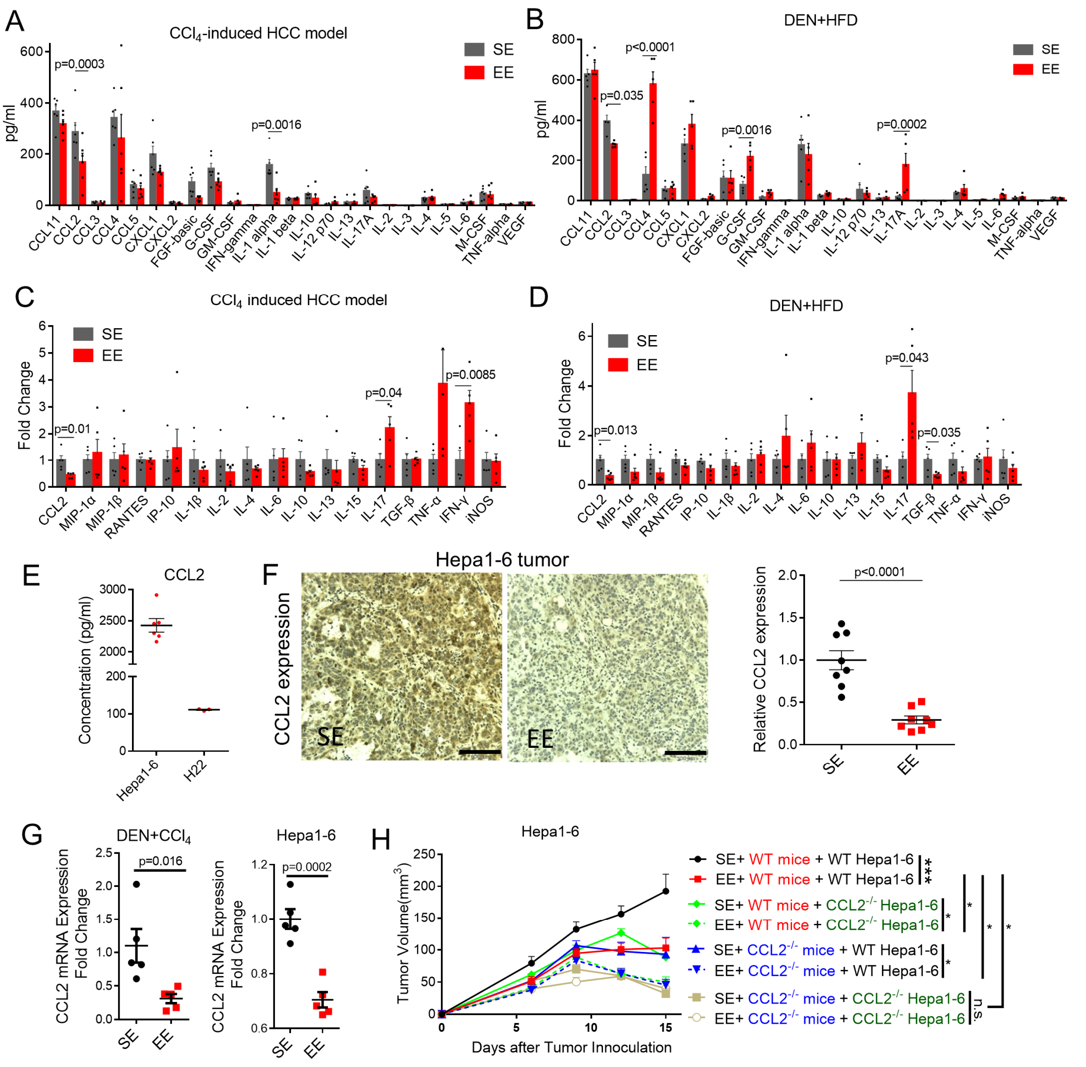

Supplementary Fig.4, related to Fig.3. CCL2 is required for the EE induced antitumor immunity

(A, B) Cytokine and chemokine profiles in serum from CCl_4-induced tumor-bearing mice (A, n=6) and DEN+HFD-induced tumor-bearing mice (B, n=5) feeding under SE or EE conditions. All data are presented as the mean ± SEM, and analyzed by two-way ANOVA.

(C, D) qPCR analysis of cytokines and chemokines mRNA expression in tumors tissues of CCl_4-induced (C) and DEN+HFD-induced (D) HCC models (n=5). All data are presented as the mean ± SEM, and analyzed by two-tailed unpaired Student's *t* test.(E) Protein level of CCL2 in cultural medium from Hepa1-6 and H22 tumor cells using ELISA assay.

(F) Representative immunostaining images of CCL2 in tumors tissue from Hepa1-6 tumor bearing mice under SE or EE feeding conditions. The relative CCL2 expression was quantified

by Image J analysis (n=8). Original magnification 20 x10, Scale bar, 100 μm. All data are presented as the mean ± SEM, and analyzed by two-tailed unpaired Student's *t* test.(G) CCL2 mRNA expression was quantified by qRT-PCR in DEN+CCl_4 induced tumors or subcutaneous Hepa1-6 tumors under SE or EE feeding condition (n=5). All data are presented as the mean ± SEM, and analyzed by two-tailed unpaired Student's *t* test.

(H) Tumor volume of subcutaneous Hepa1-6 (CCL2 WT or KO) tumors in WT or $CCL2^{-/-}$ C57BL/6 mice. Hepa1-6 cells with CCL2 KO were generated by use of CRISPR/Cas9. Hepa1-6 cells with CCL2 WT or KO were subcutaneously injected to WT or $CCL2^{-/-}$ C57BL/6 mice and fed under SE or EE conditions (SE+ WT mice + SgRNA Hepa1-6: n=11, EE+ WT mice + SgRNA Hepa1-6: n=12, SE+CCL2 ko mice + SgRNA Hepa1-6: n=6, EE+CCL2 ko mice + SgRNA Hepa1-6: n=7, SE+ WT mice +CCL2 ko Hepa1-6: n=12, EE+ WT mice +CCL2 ko Hepa1-6: n=10, SE+ CCL2 ko mice +CCL2 ko Hepa1-6: n=6, EE+ CCL2 ko mice +CCL2 ko Hepa1-6: n=7). All data are presented as the mean ± SEM, and analyzed by two-tailed unpaired Student's *t* test with $*p < 0.05$, $**p < 0.01$, $***p < 0.001$.

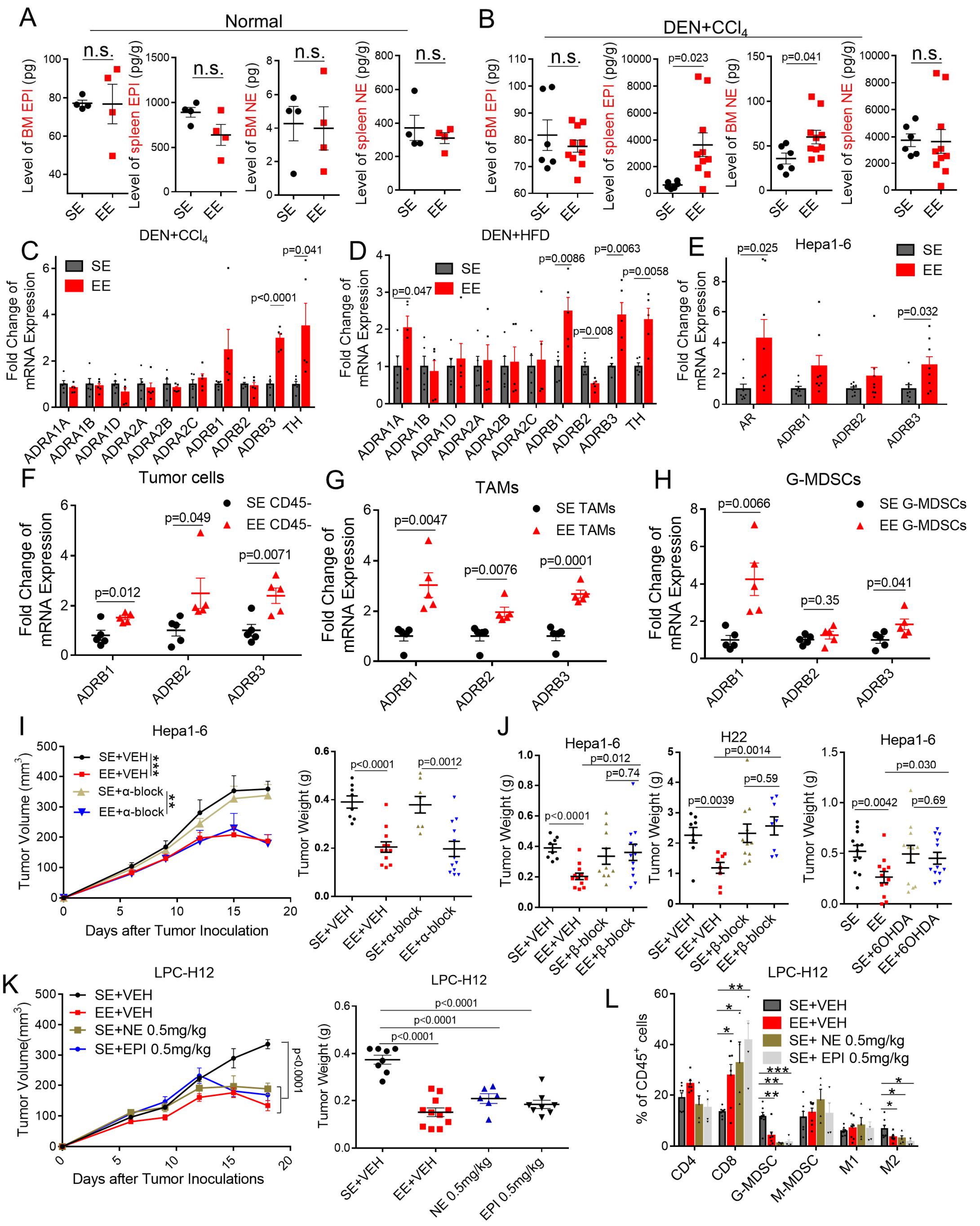

Supplementary Fig.5, related to Fig.4. EE enhances SNS/β-ARs signaling to inhibit HCC tumorigenesis and progression.

(A, B) ELISA analysis of level of EPI and NE in the spleen and bone marrow from normal mice (n=4, A) or DEN/+CCl4-treated mice (SE: n=6, EE: n=10, B) which were fed under SE or EE conductions. All data are presented as the mean ± SEM, and analyzed by two-tailed unpaired Student's *t* test.

(C, D) qPCR analysis of mRNA expression of α-ARs (ADRA1A, ADRA1B, ADRA1D,

ADRA2A, ADRA2B, ADRA2C) and β-ARs (ADRB1, ADRB2, ADRB3) and TH in tumor tissues from DEN/CCl_4-induced (A, n=5) or DEN/HFD-induced (B, n=5) HCC mouse models under SE or EE feeding conditions. All data are presented as the mean ± SEM, and analyzed by two-tailed unpaired Student's *t* test.(E) qPCR analysis of mRNA expression of total AR, ADRB1, ADRB2, and ADRB3 in tumor tissues from subcutaneous Hepa1-6 tumor-bearing mice under SE or EE feeding conditions (n=8). All data are presented as the mean ± SEM, and analyzed by two-tailed unpaired Student's *t* test. (F-H) Mostly tumor cells ($CD45^-$), TAMs cells ($CD45^+CD11b^+F4/80^+Ly6G^-$) and G-MDSCs ($CD45^+$ $CD11b^+F4/80^-Ly6G^+$) in the tumor microenvironment were sorted by flow cytometry from subcutaneous Hepa1-6 tumor-bearing mice (n=5) under SE or EE feeding conditions. mRNA expression of ADRB1, ADRB2, and ADRB3 were determined in tumor or immune cells (F), TAMs (G) and G-MDSCs (H). All data are presented as the mean ± SEM, and analyzed by two-tailed unpaired Student's *t* test.(I) Tumor volume and tumor weight of subcutaneous Hepa1-6 tumor-bearing mice treated with α-ARs blockade (α-block: Phenoxybenzamine hydrochloride, 10 mg/kg, once a week) or vehicle under SE or EE feeding conditions (n=8 for SE groups, n=12 for EE groups). All data are presented as the mean ± SEM, and analyzed by two-tailed unpaired Student's *t* test with $^{**}p<0.01$, $^{***}p<0.001$.(J) Tumor weight of Fig. 4G-I models (Hepa1-6 (left): SE+VEH n=8, EE+VEH n=12, SE+ β -block n=10, EE+ β -block n=12; H22: SE+VEH n=8, EE+VEH n=8, SE+ β -block n=11, EE+ β -block n=8; Hepa1-6 (right): SE+VEH n=12, EE+VEH n=11, SE+6OHDA n=11, EE+6OHDA n=14). All data are presented as the mean ± SEM, and analyzed by two-tailed unpaired Student's *t* test.(K) Tumor volume and tumor weight of subcutaneous LPC-H12 tumors in mice injected s.c. with 0.5mg/kg of NE or EPI every three days under SE or EE feeding conditions (n=8 for SE+VEH group and SE+EPI group, n=6 for SE+NE group, n= 11 for EE+VEH group). All data are presented as the mean ± SEM, and analyzed by two-tailed unpaired Student's *t* test.(L) Infiltrated immune cells in tumor microenvironment were analyzed by flow cytometry from LPC-H12 tumors (n=7 for SE+VEH group and EE+EPI group, n=4 for SE+NE group and SE+EPI group).. All data are presented as the mean ± SEM, and analyzed by two-tailed unpaired Student's *t* test with *, $p<0.05$; **, $p<0.01$; ***, $p<0.001$.

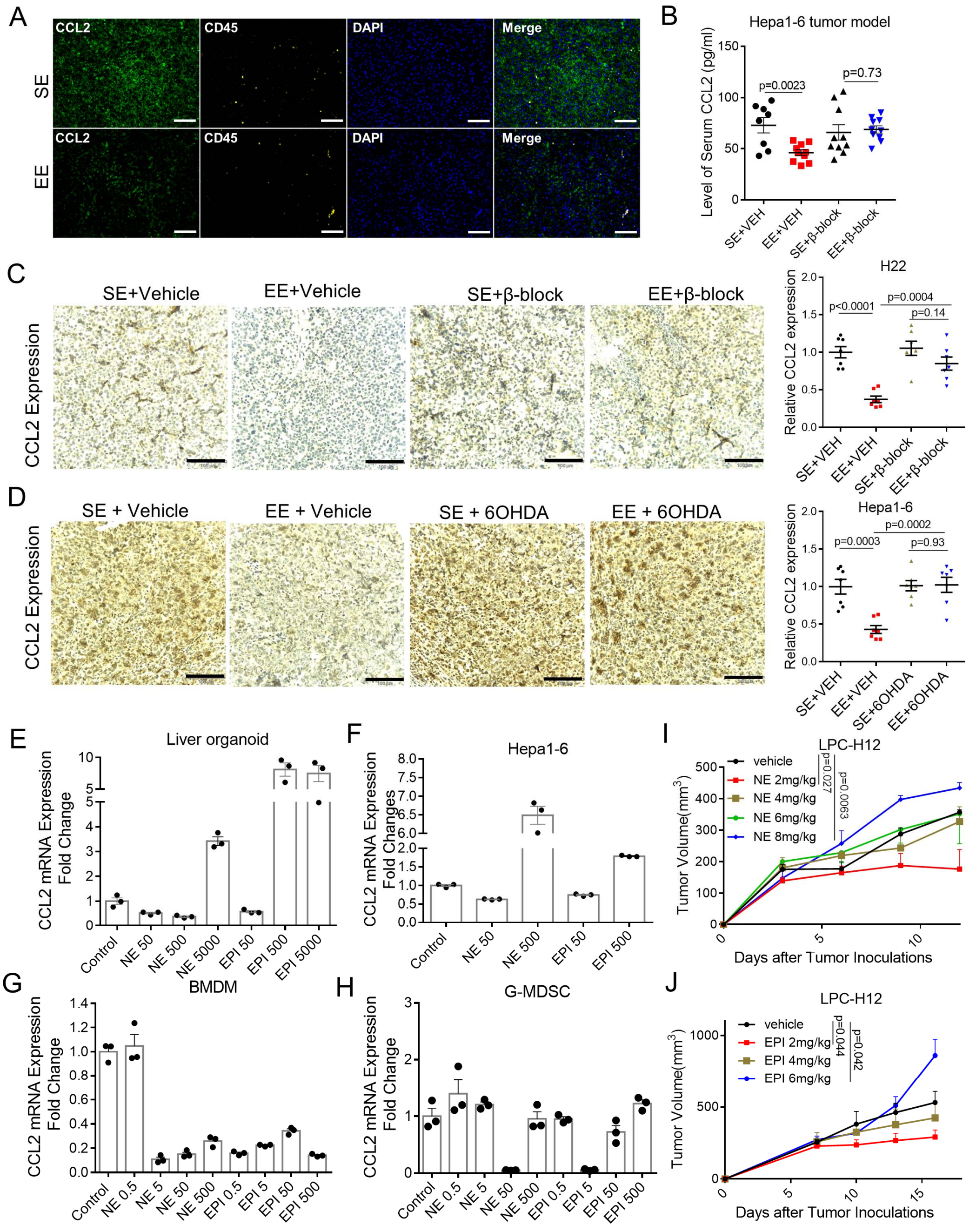

Supplementary Fig.6, related to Fig.5. Blockade of SNS/β-ARs signaling abolishes EE-induced CCL2 reduction in tumor cells and immune cells

(A) Immunofluorescence staining of CCL2, CD45 and DAPI in liver tumor tissues from DEN+CCl_4-induced tumor-bearing mice under SE or EE feeding conditions. Original magnification 20 x10, Scale bar, 100 μm.

(B) Level of CCL2 in serum from Hepa1-6 tumor-bearing mice under SE or EE feeding condition with or without β-AR blockade (SR59230A+ propranolol, n=8, for SE+VEH group, N=10, for other groups). All data are presented as the mean ± SEM, and analyzed by two-tailed

unpaired Student's *t* test.(C) Immunohistochemical staining of CCL2 in the tumor tissue from H22 tumor-bearing mice with SE or EE feeding condition with or without β-AR blockade (SR59230A+ propranolol). The relative CCL2 expression was quantified by Image J analysis (n=7). Original magnification 20 x10, Scale bar, 100 μm. All data are presented as the mean ± SEM, and analyzed by two-tailed unpaired Student's *t* test.(D) Immunohistochemical staining of CCL2 in the tumor tissue from Hepa1-6 tumor-bearing mice with SE or EE feeding condition with or without 6OHDA treatment. The relative CCL2 expression was quantified by Image J analysis (n=7). Original magnification 20 x10, Scale bar, 100 μm. All data are presented as the mean ± SEM, and analyzed by two-tailed unpaired Student's *t* test with $*p<0.05$, $**p<0.01$, $***p<0.001$.

(E) Human hepatocytes/hepatic stellate cells organoids were treated with varied concentration of NE and EPI (ng/ml) in vitro for 24h, followed by washing and medium replacement. 48 h later, CCL2 mRNA expression was determined with qPCR assay with β-actin as an internal control (n=3).

(F) The mRNA expression of CCL2 was determined with qPCR assay in Hepa1-6 cells after a 24h-treatment with varied concentration of NE and EPI (50 or 500 ng/ml) in vitro (n=3).

(G) Bone marrow derived macrophages (BMDMs) were exposed to conditioned medium (CM) of Hepa1-6 tumor cells for 48h and subsequently treated with vehicle, NE and EPI (0.5, 5, 50, 500 ng/ml) in vitro for 24h. Cells were washed followed by medium replacement. 48h later, the mRNA expressions of CCL2 in BMDM were determined

(H) Bone marrow cells were isolated from normal C57BL/6 mice and cultured in the presence of recombinant murine granulocyte macrophage colony-stimulating factor (GM-CSF) for up to 7 days. Ly6G^{+} G-MDSCs were sorted out and subsequently treated with vehicle, NE and EPI (0.5, 5, 50, 500 ng/ml) in vitro. The mRNA expressions of CCL2 in G-MDSCs were determined after treatment (n=3).

(I, J) Tumor volume of subcutaneous LPC-H12 tumors in mice injected s.c. with different doses of NE or EPI every three days under SE feeding conditions (I, n=4, for vehicle, NE 2mg/kg and 4mg/kg groups; n=6, for NE 6mg/kg and NE 8mg/kg; L, n=5, for vehicle; n=4, for EPI 2mg/kg, EPI 4mg/kg and EPI 6mg/kg). All data are presented as the mean ± SEM, and analyzed by two-tailed unpaired Student's *t* test.

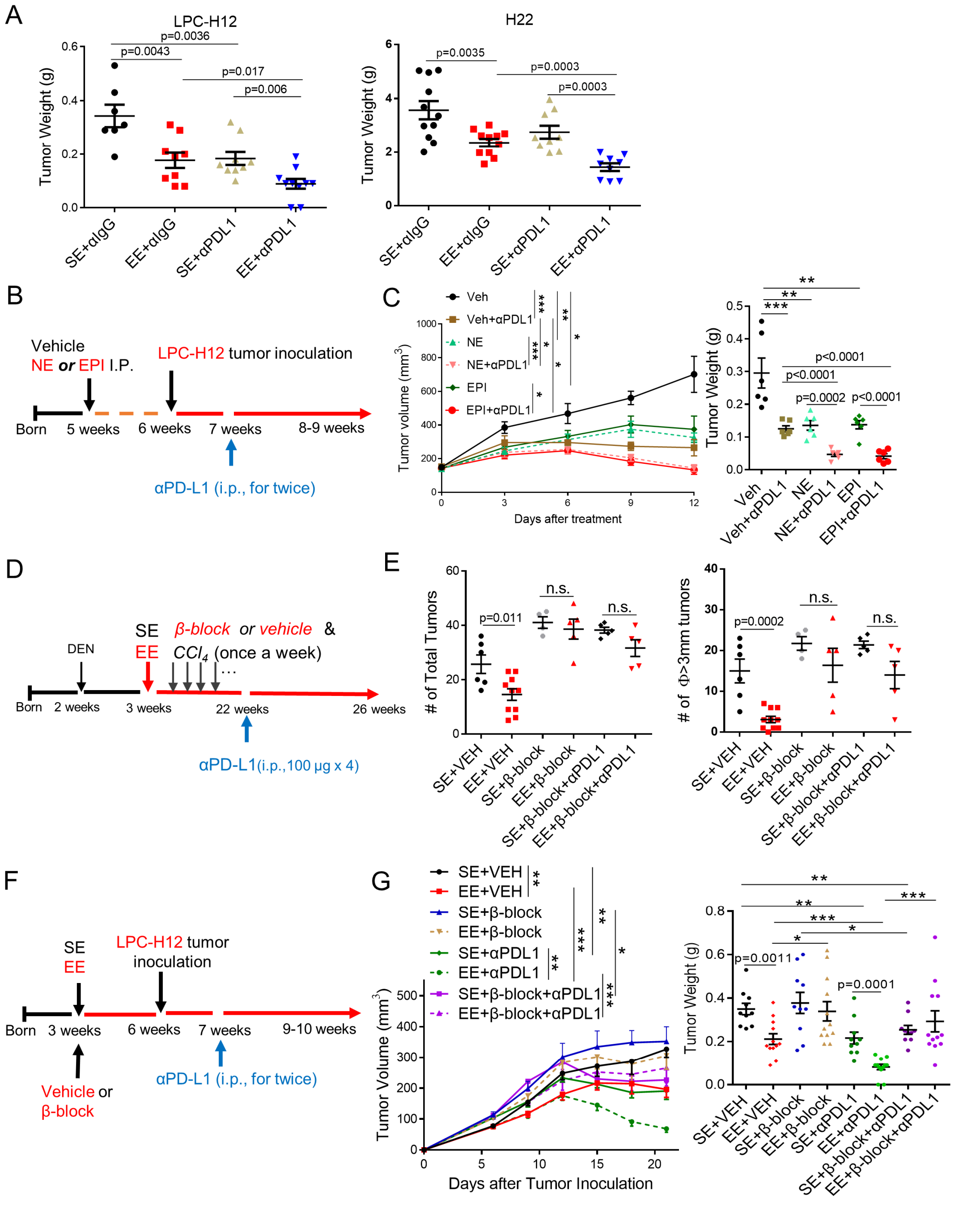

Supplementary Fig. 7, related to Fig. 6. Environmental eustress enhances tumor β-AR signaling to augment the therapeutic of PD-L1/PD-1 blockade.

(A) Tumor weight of C57BL/6 mice bearing subcutaneous LPC-H12 (H, SE+αIgG: n=7, EE+αIgG: n=9, SE+αPD-L1: n=9, EE+αPD-L1: n=10) or BALB/c mice bearing H22 tumor (I, SE+αIgG n=11, EE+αIgG n=11, SE+αPD-L1 n=9, EE+αPD-L1 n=9) treated with vehicle or anti-PD-L1 antibody under SE or EE feeding conditions. All data are presented as the mean ± SEM, and analyzed by two-tailed unpaired Student's *t* test.(B) Scheme of experimental

procedure for subcutaneous LPC-H12 tumor model with NE or EPI treatment combined with anti-PD-L1 immunotherapy.

(C) Tumor volume and tumor weight of C57BL/6 mice bearing subcutaneous LPC-H12 tumors with NE or EPI treatment plus anti-PD-L1 immunotherapy (n=6). All data are presented as the mean ± SEM, and analyzed by two-tailed unpaired Student's *t* test with $^{*}p < 0.05$, $^{**}p < 0.01$, $^{***}p < 0.001$.(D) Scheme of experimental procedure for DEN+CCl_4-induced tumor model with or without anti-PD-L1 immunotherapy, or/and β-ARs blockade treatment (β-block: SR59230A+propranolol).

(E) Total tumor numbers (left) and numbers of tumor with diameter ø≥3mm (right) on livers from DEN+CCl_4-induced tumor-bearing mice with or without anti-PD-L1 immunotherapy, or/and β-ARs blockade treatment (n=4, for SE+VEH group; n=10, for EE+VEH group; n=4, for SE+β-block group; n=5, for other groups). All data are presented as the mean ± SEM, and analyzed by two-tailed unpaired Student's *t* test.(F) Scheme of experimental procedure for subcutaneous LPC-H12 tumor model treated with or without anti-PD-L1 immunotherapy, or/and β-ARs blockade.

(G) Tumor volume and tumor weight of C57BL/6 mice bearing subcutaneous LPC-H12 tumors treated with or without anti-PD-L1 immunotherapy, or/and β-ARs blockade (n=10, for mice in SE conduction; n=12, for mice in EE conduction). All data are presented as the mean ± SEM, and analyzed by two-tailed unpaired Student's *t* test with $^{*}p < 0.05$, $^{**}p < 0.01$, $^{***}p < 0.001$.

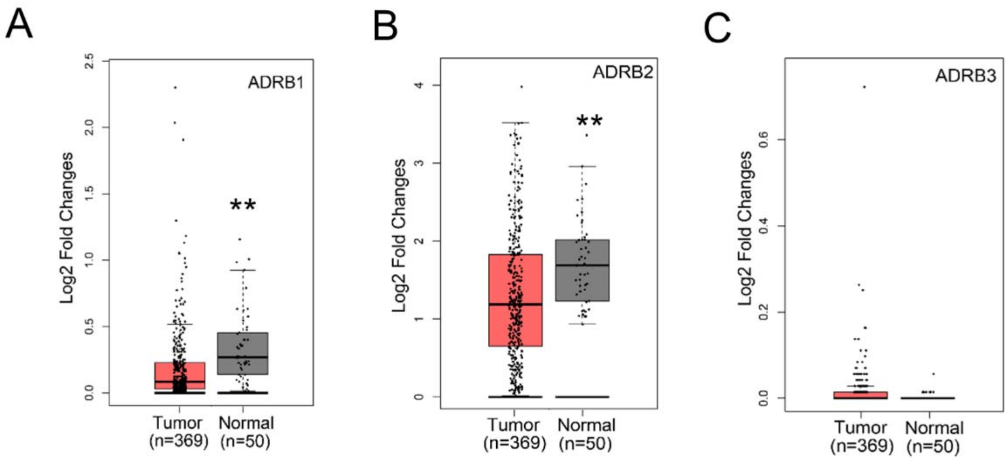

Supplementary Fig. 8. ADRB1, ADRB2 and ADRB3 mRNA expression in human liver cancer.

(A-C) Gene expression of ADRB1 (A), ADRB2 (B) and ADRB3 (C) between tumor and normal tissues in TCGA HCC samples from GEPIA web server. Data were shown as Box plot (n=369 for HCC tumor tissues; n=50 for normal liver tissue). * P<0.05 and **, P<0.01 by two-tailed unpaired Student's t test.

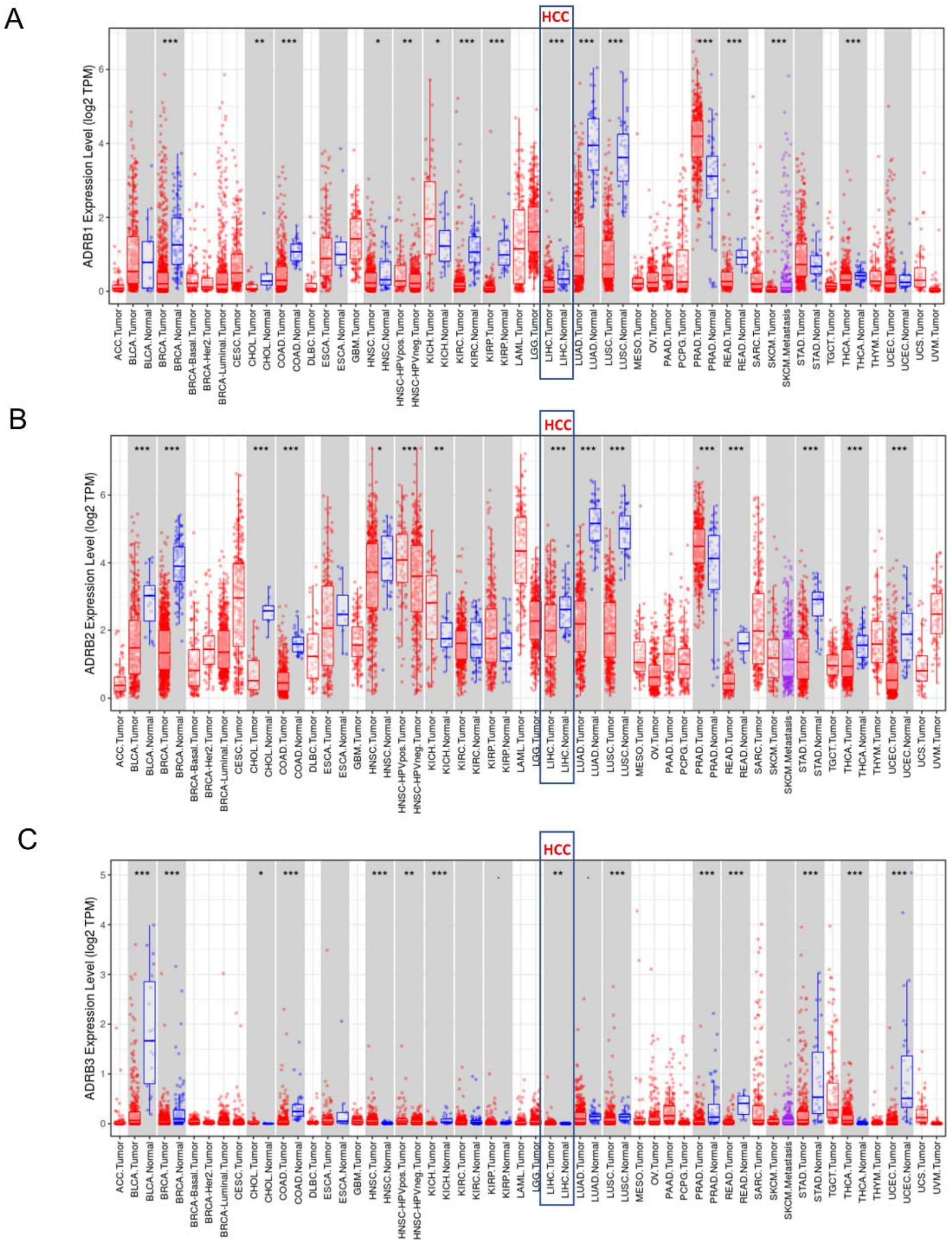

Supplementary Fig. 9. ADRB1, ADRB2 and ADRB3 mRNA expression in multiple types of human tumor and adjacent normal tissues.

Gene expression of ADRB1 (A), ADRB2 (B) and ADRB3 (C) between tumor and adjacent normal tissues across all TCGA tumors. TIMER (Tumor IMmune Estimation Resource) web server and DiffExp module were used to study the differential expression of β-ARs in diverse cancer types. Distributions of gene expression levels are displayed using box plots, with statistical significance of differential expression evaluated using the Wilcoxon test (*, $P<0.05$; **, $P<0.01$; ***, $P<0.001$). Genes that are up- or down- regulated in the tumors compared to

normal tissues for each cancer type were displayed in gray columns when normal data are available. The gene expression in HCC was indicated with the rectangular frame (n=371 for liver cancer tissues, n=50 for normal liver tissues).

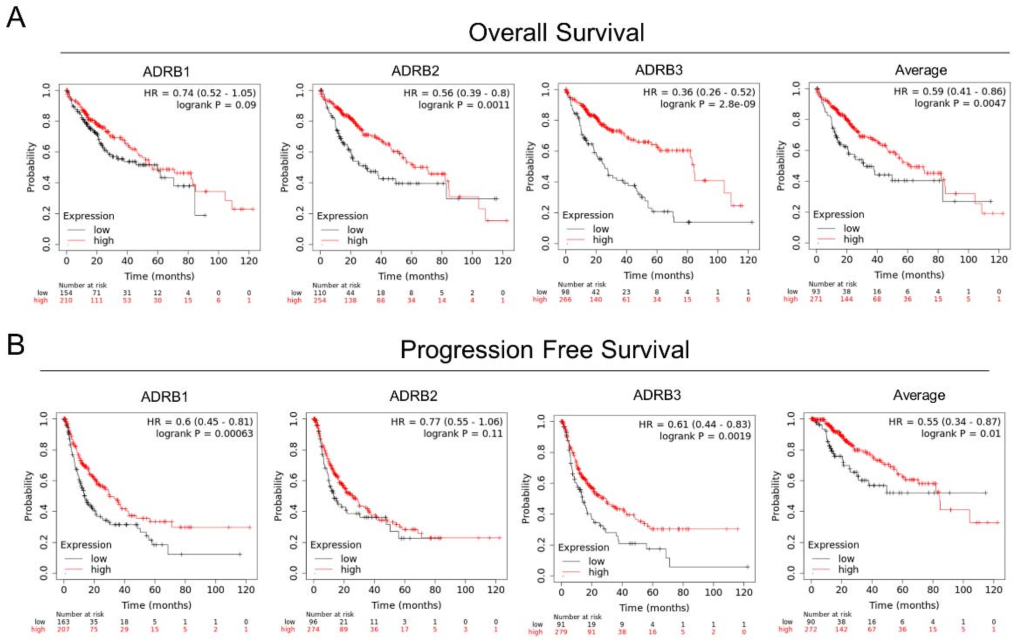

Supplementary Fig.10. Higher expression of ADRB1, ADRB2 and ADRB3 in tumor tissue is related to better prognosis in HCC patients

(A, B) The Overall Survival (A, n=364) and Progression Free Survival (B, n=370) of patients with HCC was compared between individuals bearing tumors with high or low levels of ADRB1, ADRB2, ADRB3, total ADRB (ADRB1+ADRB2+ADRB3) mRNA transcription, as measured by RNA-seq. The datasets were from the Kaplan-Meier Plotter database. Hazard ratio, 95% confidence interval and P values from the log-rank test are shown.

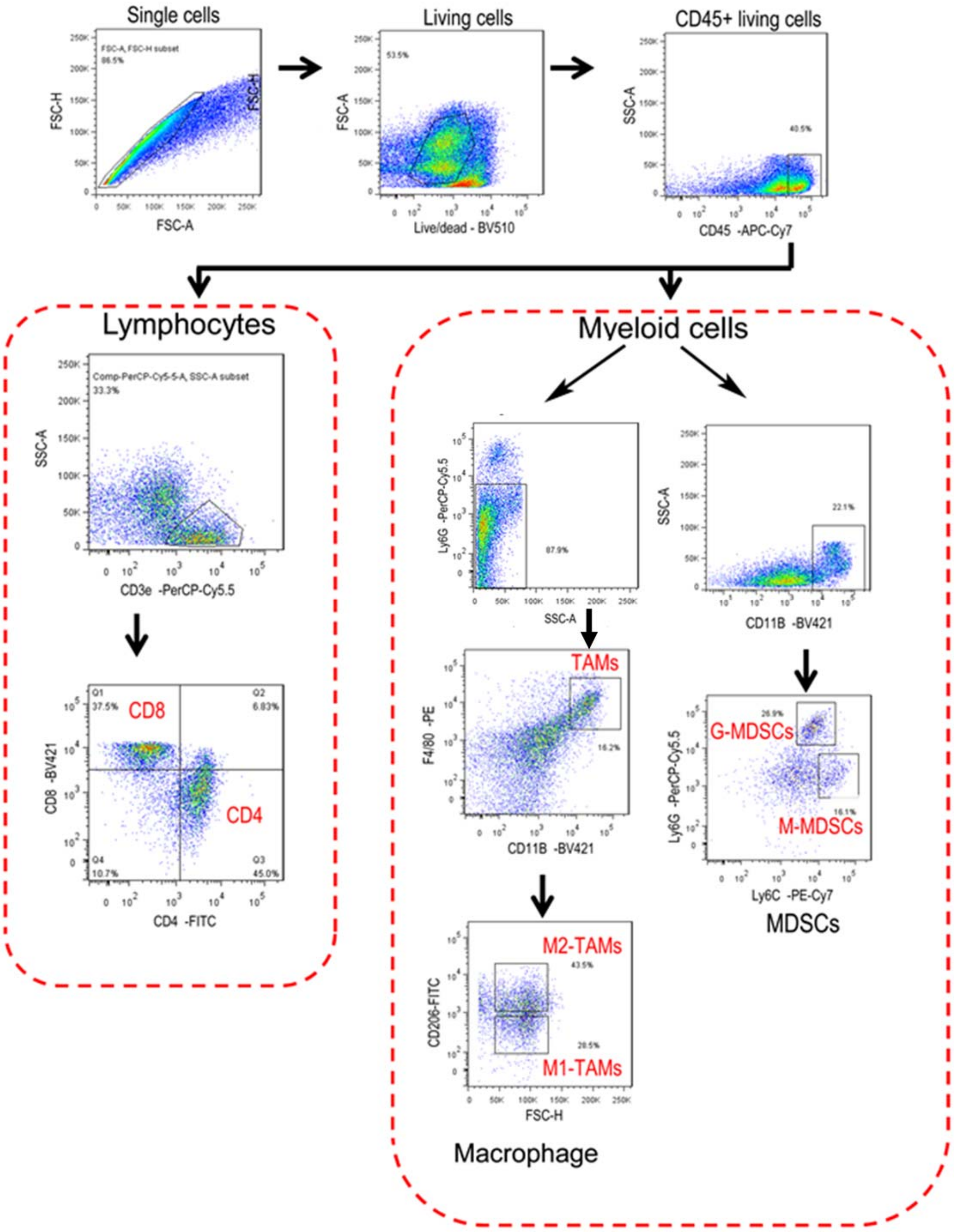

Single cells
Living cells
CD45+ living cells
FSC-H
FSC-A
FSC-A
Live/dead - BV510
SSC-A
CD45 -APC-Cy7
Lymphocytes
Myeloid cells
SSC-A
CD3e -PerCP-Cy5.5
CD8
CD4
CD8 -BV421
CD4 -FITC
Ly6G -PerCP-Cy5.5
SSC-A
CD11B -BV421
TAMs
F4/80 -PE
CD11B -BV421
G-MDSCs
M-MDSCs
Ly6G -PerCP-Cy5.5
Ly6C -PE-Cy7
MDSCs
M2-TAMs
M1-TAMs
CD206-FITC
FSC-H
Macrophage

Supplementary Fig.11. Example of gating schematics to characterize the infiltrated immune cells in tumor tissue.

Supplementary Table 1 Reagents used in this study.

**Reagents**	**Source**	**Identifier**
Carbon Tetrachloride(CCL4)	Sibas	Cat#H1S0670
Diethylnitrosamine (DEN)	Sinopharm	Cat#73861
60 kcal% Fat (HFD)	Research Diets	Cat#D12492
Propranolol hydrochloride	Sigma	Cat#P0884
SR 59230A	Sigma	Cat#S8688
6-Hydroxydopamine hydrobromide	Sigma	Cat#162957
N-(2-Chloroethyl)-N-(1-methyl-2-phenoxyethyl) Benzylamine Hydrochloride	TCI	Cat#D0158
Epinephrine Hydrochloride	Sigma	Cat#E4642
Norepinephrine Bitartrate salt	Absin	Cat#abs42040313
Penicillin Streptomycin Sol	Gibco	Cat#15140122
DMEM, High Glucose	Gibco	11965118
RPMI 1640	ThermoFishe	Cat#C11875500CP
IMDM	ThermoFishe	Cat#12440053
Recombinant Murine M-CSF	PeproTech	Cat#31502100
Recombinant Murine GM-CSF	PeproTech	Cat#3150350
Methyl sulfoxide(DMSO)	Sigma	Cat#527963
Mouse Magnetic Luminex Screening Assay	R&D	Cat#LXSAMSM
Human CCL2 ELISA Kit	Sino Biological	Cat#KIT10134
Mouse CCL2 ELISA Kit	Abcam	Cat#ab100721
Epinephrine/Norepinephrine ELISA Kit	Abnova	Cat#KA3768
Mouse MDSCs Negative Selection Kit	Stemcell	Cat#19762
Ms IFN-Gma CBA Flex Set A4	BD Pharmingen	Cat#558296
Mouse IFN gamma PicoKine ELISA Kit	Boster	Cat#EK0375
OpalTM 7-Color Manual IHC Kit	Perkinelmer	Cat#NEL811001KT

Supplementary Table 2 Antibodies used in this study.

**Antibodies**	**SOURCE**	**IDENTIFIER**
**Antibodies for flow cytometry**		
Fixable Viability Stain 510	BD Pharmingen	Cat#564406
Ms CD45 APC-Cy7	BD Pharmingen	Cat#557659
Ms CD3e PerCP-Cy5.5	BD Pharmingen	Cat#551163
Ms CD4 FITC	BD Pharmingen	Cat#553046
Ms CD8a BV421	BD Pharmingen	Cat#563898
Ms CD11B BV421	BD Pharmingen	Cat#562605
Ms F4/80 PE	BD Pharmingen	Cat#565410
Ms CD16/CD32 Pure	BD Pharmingen	Cat#553141
Ms CD206 FITC	Biolegend	Cat#141704
Ms Ly-6G/Ly-6C FITC	BD Pharmingen	Cat#553127
Ms Ly-6G PerCP-Cy5.5	BD Pharmingen	Cat#560602
Ms Ly-6C PE-Cy7	BD Pharmingen	Cat#560593
Ms NK-1.1 PE	BD Pharmingen	Cat#553165
**Antibodies for IHC/ IF**		
Rabbit Anti-CD8 antibody	Abcam	Cat#ab203035
Rabbit Anti-F4/80 antibody	Abcam	Cat#ab100790
Rabbit Anti-CD45 antibody	Abcam	Cat#ab10558
Rabbit Anti-Ly6C antibody	Abcam	Cat#ab15627
Rabbit Anti-Ly6G antibody	Abcam	Cat#ab25377
Rabbit Anti-MCP1 antibody	Abcam	Cat#ab25124
Rabbit Anti-ADRB1 Polyclonal Antibody	Absin	Cat#abs119982a
Rabbit Anti-ADRB2 Polyclonal Antibody	Absin	Cat#abs120301a
Rabbit Anti-ADRB3 Polyclonal Antibody	Absin	Cat#abs120618a
**Antibodies for Western Blotting**		
Anti-MCP1 antibody	Abcam	Cat#ab9899
GAPDH (14C10) Rabbit mAb	Cell Signaling	Cat#2118L
Antibodies for *in vivo* cell depletion, CCL2 neutralization and immunotherapy		
InVivoMab anti-mouse CD8 (53-6.7)	BioXcell	Cat#BE0004-1
InVivoMab anti-mouse CD4 (GK1.5)	BioXcell	Cat#BE0003-1
InVivoMab anti-mouse Ly-6G (1A8)	BioXcell	Cat#BE0075-1
InVivoMAb anti-mouse NK1.1 (PK136)	BioXcell	Cat# BE0036
InVivoMab anti-mouse CCL2 (2H5)	BioXcell	Cat#BE0185
InVivoMab anti-mouse PD-L1 (10F.9G2)	BioXcell	Cat#BE0101
InVivoMAb rat IgG2a isotype control, anti-trinitrophenol	BioXcell	Cat#BE0089
InVivoMAb rat IgG2b isotype control, anti-keyhole limpet hemocyanin	BioXcell	Cat#BE0090
InVivoMAb polyclonal Armenian hamster IgG	BioXcell	Cat#BE0091

Supplementary Table 3 Primers used in this study.

**Oligonucleotides**
Mouse 18S rRNA forward primer: GAGCGAAAGCATTTGCCAAG
Mouse 18S rRNA reverse primer: GGCATCGTTTATGGTCGGAA
Mouse β-actin forward primer: AGAGGGAAATCGTGCGTGAC
Mouse β-actin reverse primer: CAATAGTGATGACCTGGCCGT
Mouse CCL2 forward primer: TTAAAAACCTGGATCGGAACCAA
Mouse CCL2 reverse primer: GCATTAGCTTCAGATTTACGGGT
Mouse MIP-1α forward primer: TTCTCTGTACCATGACACTCTGC
Mouse MIP-1α reverse primer: CGTGGAATCTTCCGGCTGTAG
Mouse MIP-1β forward primer: TTCCTGCTGTTTCTCTTACACCT
Mouse MIP-1β reverse primer: CTGTCTGCCTCTTTTGGTCAG
Mouse RANTES forward primer: TTACCAGCACAGGATCAAATGG
Mouse RANTES reverse primer: CGGAAGTAGAATCTCACAGCAC
Mouse IP-10 forward primer: CCAAGTGCTGCCGTCATTTTC
Mouse IP-10 reverse primer: GGCTCGCAGGGATGATTTCAA
Mouse IL-1β forward primer: GCAACTGTTCCTGAACTCAACT
Mouse IL-1β reverse primer: ATCTTTTGGGGTCCGTCAACT
Mouse Il2 forward primer: TGAGCAGGATGGAGAATTACAGG
Mouse Il2 reverse primer: GTCCAAGTTCATCTTCTAGGCAC
Mouse Il4 forward primer: GGTCTCAACCCCCAGCTAGT
Mouse Il4 reverse primer: GCCGATGATCTCTCTCAAGTGAT
Mouse Il6 forward primer: TAGTCCTTCCTACCCCAATTTCC
Mouse Il6 reverse primer: TTGGTCCTTAGCCACTCCTTC
Mouse Il10 forward primer: GCTCTTACTGACTGGCATGAG
Mouse Il10 reverse primer: CGCAGCTCTAGGAGCATGTG
Mouse Il13 forward primer: CCTGGCTCTTGCTTGCCTT
Mouse Il13 reverse primer: GGTCTTGTGTGATGTTGCTCA
Mouse Il15 forward primer: ACATCCATCTCGTGCTACTTGT
Mouse Il15 reverse primer: GCCTCTGTTTTAGGGAGACCT
Mouse Il17 forward primer: CACCCCCGGAACACCAAAG
Mouse Il17 reverse primer: CATACTCTTCCATTCGAGCGTAG
Mouse TGF-β forward primer: GCTAATGGTGGACCGCAACAAC
Mouse TGF-β reverse primer: CACTGCTTCCCGAATGTCTGAC
Mouse TNF-α forward primer: GCGACGTGGAACTGGCAGAAGAG
Mouse TNF-α reverse primer: TGAGAGGGAGGCCATTTGGGAAC
Mouse IFN-γ forward primer: ATGAACGCTACACACTGCATC
Mouse IFN-γ reverse primer: CCATCCTTTTGCCAGTTCCTC
Mouse iNOS forward primer: CTGCTGGTGGTGACAAGCACATTT
Mouse iNOS reverse primer: ATGTCATGAGCAAAGGCGCAGAAC
Mouse ADRA1A forward primer: CTGCCATTCTTCCTCGTGAT
Mouse ADRA1A reverse primer: GCTTGGAAGACTGCCTTCTG

Mouse ADRA1B forward primer: AACCTTGGGCATTGTAGTCG
Mouse ADRA1B reverse primer: CTGGAGCACGGGTAGATGAT
Mouse ADRA1D forward primer: TCCGTAAGGCTGCTCAAGTT
Mouse ADRA1D reverse primer: CTGGAGCAGGGGTAGATGAG
Mouse ADRA2A forward primer: TGCTGGTTGTTGTGGTTGTT
Mouse ADRA2A reverse primer: GGGGGTGTGGAGGAGATAAT
Mouse ADRA2B forward primer: GCCACTTGTGGTGGTTTTCT
Mouse ADRA2B reverse primer: TTCCCCAGCATCAGGTAAAC
Mouse ADRA2C forward primer: TCATCGTTTTCACCGTGGTA
Mouse ADRA2C reverse primer: GCTCATTGGCCAGAGAAAAG
Mouse ADRB1 forward primer: TCGCTACCAGAGTTTGCTGA
Mouse ADRB1 reverse primer: GGCACGTAGAAGGAGACGAC
Mouse ADRB2 forward primer: GACTACACAGGGGAGCCAAA
Mouse ADRB2 reverse primer: TGTCACAGCAGAAAGGTCCA
Mouse ADRB3 forward primer: TGAAACAGCAGACAGGGACA
Mouse ADRB3 reverse primer: TCAGCTTCCCTCCATCTCAC
Mouse TH forward primer: GTCTCAGAGCAGGATACCAAGC
Mouse TH reverse primer: CTCTCCTCGAATACCACAGCC
Mouse AR forward primer: GTCTCAGAGCAGGATACCAAGC
Mouse AR reverse primer: CTCTCCTCGAATACCACAGCC
Human β-actin forward primer: AAGGAGCCCCACGAGAAAAT
Human β-actin reverse primer: ACCGAACTTGCATTGATTCCAG
Human CCL2 forward primer: CATCTCCTACACCCCACGAAG
Human CCL2 reverse primer: GGGTTGGCACAGAAACGTC
